# Recent Developments in the Reformatsky-Claisen Rearrangement

**DOI:** 10.3390/molecules171214249

**Published:** 2012-11-30

**Authors:** Jun Ishihara, Susumi Hatakeyama

**Affiliations:** Graduate School of Biomedical Sciences, Nagasaki University, 1-14 Bunkyo-machi, Nagasaki 852-8521, Japan; E-Mail: susumi@nagasaki-u.ac.jp

**Keywords:** Reformatsky-Claisen rearrangement, Reformatsky reaction, Claisen rearrangement, zinc, indium

## Abstract

The rearrangement of allyl α-bromoacetates with Zn dust is known as the Reformatsky-Claisen rearrangement. Whereas the Ireland-Claisen rearrangement has been widely used in the synthesis of a diverse range of natural products, the Zn-mediated Reformatsky-Claisen rearrangement has not been utilized so often. In this article, we will provide an overview of recent advances in the Reformatsky-Claisen rearrangement field, including the In-mediated Reformatsky-Claisen rearrangement we have recently developed.

## 1. Introduction

The development of new methods for stereoselective carbon-carbon bond formation has been important in the creation of useful molecules such as drugs and other chemical entities. [3,3]-Sigmatropic rearrangements are reliable reactions for selective carbon-carbon bond formation, in particular, the Claisen rearrangement is one of the most competent methods to provide useful building blocks for the synthesis of natural products [1,2,3,4]. The synthetic utility of this reaction has prompted the development of a considerable number of variants of the classical Claisen rearrangement [5,6,7,8,9]. For instance, zinc-mediated [3,3]-sigmatropic rearrangement of α-haloesters, proceeding through Zn enolates, are referred to as the Reformatsky-Claisen rearrangement. Compared to the Ireland-Claisen rearrangement which was widely used in the synthesis of a diverse range of natural products [10,11,12,13,14], the Reformatsky-Claisen rearrangement has the advantage of being performed under non-basic conditions. This review article focuses on the chemistry of the Reformatsky-Claisen rearrangement and its applications, as well as the recent development of the In-mediated Reformatsky-Claisen rearrangement.

## 2. Pioneering Works of the Reformatsky-Claisen Rearrangement

**Scheme 1:**
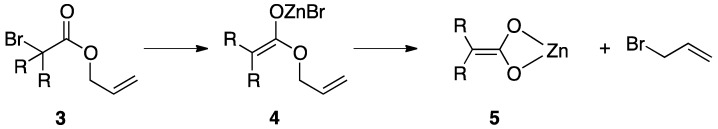
Possible pathway to generate byproduct.

In 1973, Baldwin and Walker reported a synthetically useful sigmatropic rearrangement of α-halogenated allyl esters [15]. Zinc enolates, generated by Reformatsky-type reactions of α-halogenated allyl esters with zinc dust, provided the corresponding rearranged products. For instance, when allyl α-bromoisobutyrate **1a** was added to a refluxing suspension of an excess amount of Zn dust it afforded acid **2a** in excellent yield (Table 1). It should be noted that the rearrangement of **1a** can readily install a quaternary center into the product. The reaction of α-bromopropionate **1b** also proceeded smoothly to give a rearranged product **2b**. In contrast, the rearrangement of simple allyl ester **1c** and secondary ester **1d** were found to be fruitless. Baldwin stated that one of the reasons for the low yield would be the generation of 1,3-dicarbonyl products by intermolecular Claisen condensation, which had been known as byproducts of classical Reformatsky reactions. Another reason would arise from the decomposion of Zn enolate **4** to afford **5** and allyl bromide (Scheme 1). The resulting allyl bromide would undergo Friedel-Crafts reaction with the aromatic solvent catalyzed by the zinc bromide generated in the reaction. 

**Table 1:** Baldwin and Walker’s pioneering work of the Reformatsky-Claisen rearrangement.

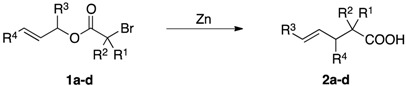							
Substrate	R^1^	R^2^	R^3^	R^4^	Solvent	Temp (°C)	Yield of 2a–d
**1a**	Me	Me	H	H	PhH	80	100%
**1b**	Me	H	H	Me	PhMe	110	96%
**1c**	H	H	H	H	Xylene	140	<15%
**1d**	Me	H	Ph	H	PhMe	110	16%

### 2.1. Reformatsky-Claisen Rearrangement in the Presence of Zinc and a Silylating Reagent

**Scheme 2:**
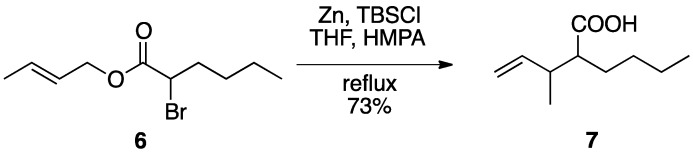
Reformatsky-Claisen rearrangement with zinc and a silylating reagent.

The Reformatsky-Claisen rearrangement also proceeds in the presence of a silylating agent, in which a silyl ketene acetal is the most likely intermediate. Ireland and co-workers demonstrated that upon heating a mixture of α-bromo ester **6**, Zn dust, and TBSCl in THF and HMPA under reflux, the carboxylic acid **7** was obtained in 73% yield (Scheme 2) [11]. This reaction would be evaluated as a base-free reaction complementary to the ester enolate Claisen rearrangement.

**Scheme 3:**
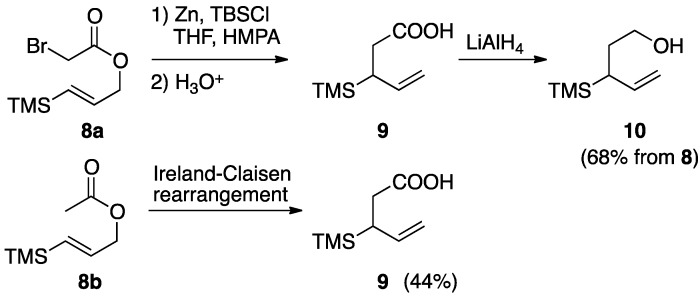
Reformatsky-Claisen rearrangement of **8a**.

An example of a reactions performed under these conditions was illustrated by Akiba, which involves preparation of a carboxylic acid bearing a silyl group [16]. A thermal reaction of α-bromoacetate **8a** with Zn and TBSCl in THF and HMPA provided carboxylic acid **9**, LiAlH_4_ reduction of which afforded alcohol **10** in 68% overall yield. On the other hand, application of the Ireland-Claisen protocol to acetate **8b** resulted in the production of the same carboxylic acid **9** in lower yield (Scheme 3).

**Scheme 4:**
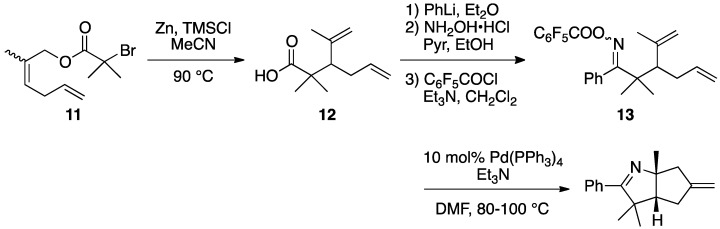
Preparation of functionalized carboxylic acid **12**.

Narasaka and co-workers employed the Reformatsky-Claisen rearrangement for the preparation of highly functionalized carboxylic acid **12**. Exposure of α-bromoisobutyrate **11** to Zn and TMSCl furnished branched acid **12,** having a quaternary carbon. The product **12** was transformed to a dienyl oxime **13**, which was a precursor in a palladium-catalyzed domino cyclization (Scheme 4) [17].

### 2.2. Application of Reformatsky-Claisen Rearrangement with Zinc and a Silylating Reagent

The most frequently reported Reformatsky-Claisen protocol involves heating a substrate with Zn dust and a silylating reagent in an aprotic polar solvent. Several additional applications are described below. 

#### 2.2.1. Preparation of α-Fluorocarboxylic Acid by Reformatsky-Claisen Rearrangement

**Scheme 5:**
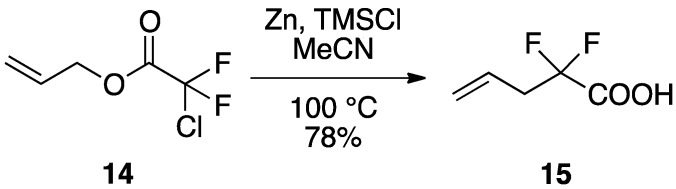
Reformatsky-Claisen reaction of fluorinated substrate **14**.

Fluorinated ketones have been successfully employed as enzyme inhibitors in modern bioorganic chemistry. Therefore, the synthesis of selectively fluorinated molecules which have fluorine substituents adjacent to a carbonyl group became a major target in fluoroorganic chemistry. One of the earliest applications of Reformatsky-Claisen rearrangement of fluorinated substrates, reported by Lang and co-workers, was the conversion of allyl chlorodifluoroacetate **14** to difluoroacid **15** (Scheme 5). Various allyl chlorodifluoroacetates can undergo a Reformatsky-Claisen protocol to give 2,2-difluoro-4-pentenoic acid derivatives [18].

#### 2.2.2. Application of α-Fluorocarboxylic Acid Induced by Reformatsky-Claisen Rearrangement to Biological Active Compounds

**Scheme 6:**
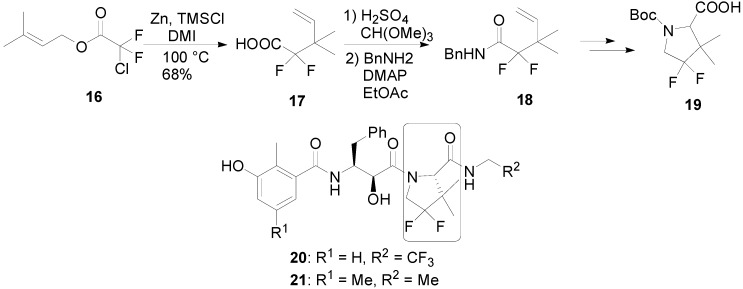
Synthesis of 2,2-Difluoroproline Derivative by Reformatsky-Claisen Rearrangement.

An intriguing extension of this methodology would be found in the synthesis of a key building block for a number of second-generation HIV protease inhibitors reported by Chen’s group at Pfizer. They performed the reaction of chlorodifluoro derivative **16** with Zn and TMSCl in 1,3-dimethyl-imidazolidin-2-one (DMI) for the preparation of difluorocarboxylic acid **17**. The product **17** was then converted to amide **18**, from which 4,4-difluoro-3,3-dimethylproline derivative **19**, a core part of HIV protease inhibitors such as **20** and **21** was synthesized (Scheme 6) [19].

**Scheme 7:**
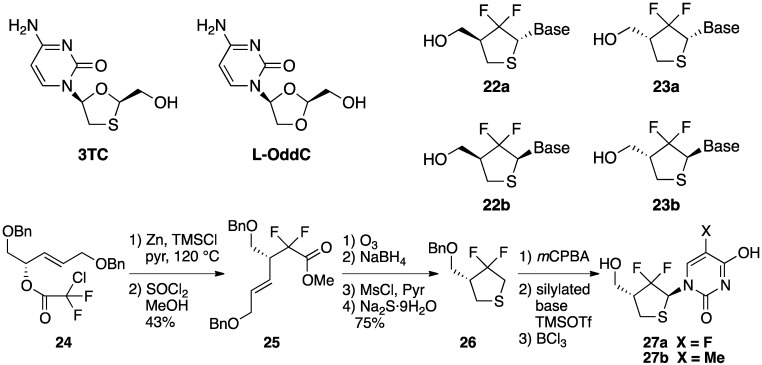
Synthesis of thionucleosides through Reformatsky-Claisen Rearrangement by Qing.

Qing and co-workers employed a Reformatsky-Claisen rearrangement to synthesize fluorinated thionucleosides. The replacement of a carbohydrate moiety of naturally occurring nucleosides with other five membered rings is one of the promising approaches for exerting a significant effect on the biologic activity. In the course of the studies of nucleoside analogues, they targeted difluoromethylene containing thionucleosides, such as **22** and **23**, which were the modified analogs of highly bioactive (−)-2'-deoxy-3'-thiacytidine (3TC) and (+)-2'-deoxy-3'-oxacytidine (L-OddC) (Scheme 7) [20]. When Reformatsky-Claisen rearrangement of **24** was carried out under conventional conditions (Zn, and TMSCl), none of desired product was obtained. On the other hand, the addition of pyridine promoted this rearrangement effectively. Thus, treatment of **24** with Zn and TMSCl in the presence of pyridine at 120 °C in a sealed tube afforded the desired product **25** in 43% yield. Compound **25** was then transformed to thiofuranose **26** in 4 steps, which was condensed with pyrimidine bases by regioselective Pummerer reaction to afford nucleosides **27a** and **27b** (Scheme 7).

**Scheme 8:**
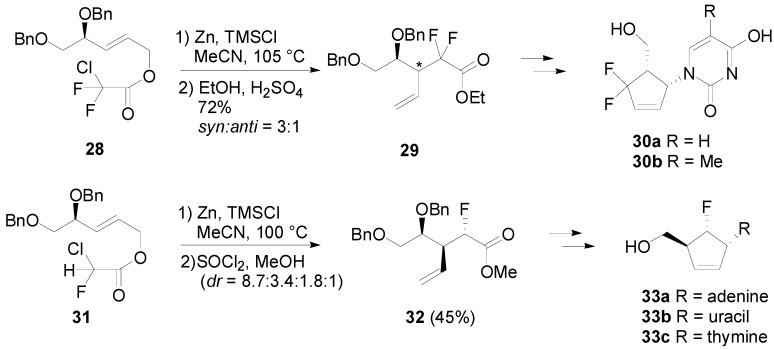
Synthesis of carbocyclic nucleosides by Qing.

Qing’s group further targeted fluorinated carbocyclic nucleosides based on the similar strategy. Upon treatment of **28** with Zn and TMSCl at 105 °C in MeCN, the rearrangement took place to deliver difluorinated acids, esterification of which afforded ethyl ester **29** as a mixture (*syn*:*anti* = 3:1) in 72% yield [21]. On the other hand, the reaction of monofluoro ester **31** provided four isomers (*dr* = 8.7:3.4:1.8:1), major component of which was *syn*-*anti*-product **32**. The resulting esters **29** and **32** were convertible to cyclopentenes **30a**,**b**, and **33a–c** after installation of nucleobases, respectively (Scheme 8) [22]. 

## 3. Indium-Mediated Reformatsky-Claisen Rearrangement

**Scheme 9:**
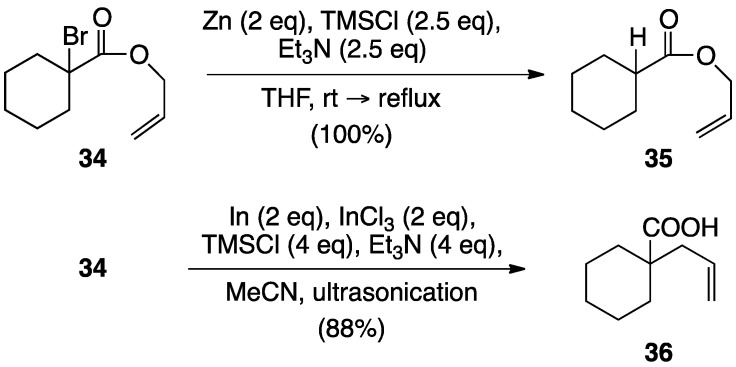
Reformatsky-Claisen rearrangement of **34**.

Recently Ishihara and Hatakeyama reported the In-mediated Reformatsky-Claisen rearrangement, which is feasible for various α-bromoisobutyrate derivatives [23,24]. Initially, α-bromo-cyclohexanecarboxylate **34** was subjected to the conventional rearrangement conditions. However, when **34** was treated with Zn and TMSCl-Et_3_N in boiling THF, the protonated product **35** was obtained exclusively (Scheme 9). The scope of the Reformatsky reaction has been considerably extended by the use of metals other than Zn. For instance, in is known to react readily with α-halo esters to induce the Reformatsky-type reaction [25]. Recently Baba and co-workers demonstrated that In(I)X is effective for the Reformatsky-type reactions of ketones and esters to afford β-hydroxyketones and β-hydroxyesters diastereoselectively [26,27,28]. Gratifyingly, we found that treatment of **34** with In-InCl_3_ in the presence of TMSCl and Et_3_N in MeCN under ultrasonication conditions at 10–30 °C furnished **36** in 88% yield.

### 3.1. Indium-Mediated Reformatsky-Claisen Rearrangement of α-Bromopropionates

**Scheme 10:**
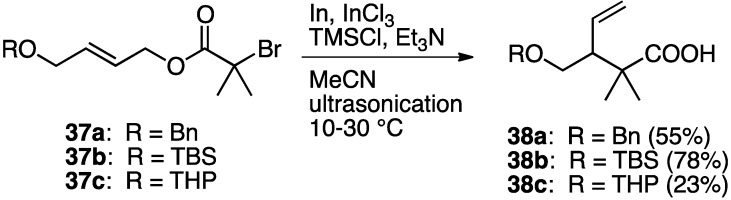
Indium-mediated Reformatsky-Claisen rearrangement.

To probe the generality of the In-mediated reaction, α-bromoesters **37a**–**c** were subjected to the optimized reaction conditions (Scheme 10). The benzyl and TBS ethers were also not affected at all under the conditions; however the THP group was susceptible owing to the Lewis acidity of InCl_3_.

### 3.2. Indium-Mediated Reformatsky-Claisen Rearrangement of Various Substrates

We next turned our attention to the reactions of various α-bromopropionate derivatives, which are readily prepared by acylation of the corresponding allylic alcohols with 2-bromoisobutyryl bromide or 2-bromopropionyl bromide (Table 2). Most reactions afforded the rearranged products along with the protonated compounds. 

**Table 2:** Indium-mediated Reformatsky-Claisen Rearrangement of Various Substrates.

Substrates	R	Method	Products		Yield
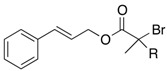	**39a**: Me	a	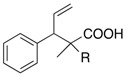	**40a**	96%
	**39b**: H	b		**40b**	84% (1.5:1)
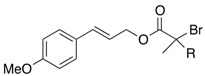	**41a**: Me	a	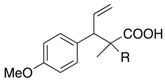	**42a**	94%
	**41b**: H	b		**42b**	61% (1.6:1)
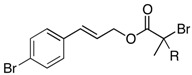	**43a**: Me	a	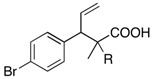	**44a**	71%
	**43b**: H	b		**44b**	54% (1.4:1)
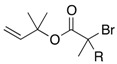	**45a**: Me	a	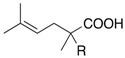	**46a**	63%
	**45b**: H	b		**46b**	62%
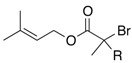	**47a**: Me	a	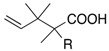	**48a**	91%
	**47b**: H	b		**48b**	34%

Method a: In (2 eq.), InCl_3_ (2 eq.), TMSCl (8 eq.), Et_3_N (8 eq.), MeCN, 10–30 °C; Method b: In (2 eq.), InCl_3_ (2 eq.), TMSCl (4 eq.), Et_3_N (4 eq.), THF-DMPU (1:1), 10–30 °C.

The aromatic compounds **39**, **41**, and **43** underwent rearrangement to carboxylic acids **40**, **42**, and **44** in moderate to excellent yields, although the diastereoselectivities were poor. In the case of **39a**, **41a**, and **43a**, the rearrangement took place in MeCN rather smoothly, whereas the rearrangement of **39b**, **41b**, and **43b** proceeded in THF-DMPU (1:1) but not in MeCN. On the other hand, the reactions of aliphatic substrates **45** and **47** brought about the Reformatsky-Claisen rearrangement successfully to give highly functionalized carboxylic acids **46** and **48**. In fact, when the In-mediated reaction of bulky 2-methylbut-3-en-2-yl esters **45a** and **45b** were performed, compounds **46a** and **46b** were obtained in 63% and 62% yields, respectively. Notably, the rearrangement of **47a** can install contiguous quaternary centers, giving compound **48a** in 91% yield.

### 3.3. The Reaction of Base-Sensitive Compounds

**Scheme 11:**
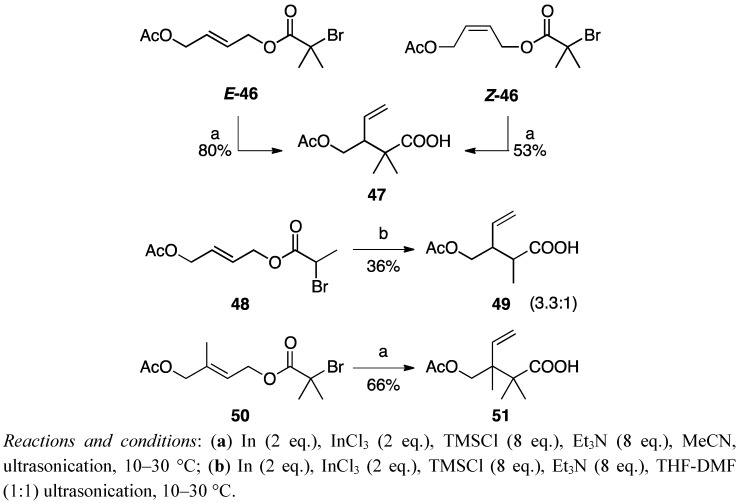
Investigation of the Reformatsky-Claisen rearrangement of acetoxy α-bromoesters.

The most intriguing feature of the Reformatsky-Claisen rearrangement is the feasibility of utilizing base-sensitive substrates. The reactions of α-bromoisobutyrates E-49 and Z-49 under the optimized conditions proceeded smoothly to afford 50 in 80% and 53% yields, respectively (Scheme 11). In the case of α-bromopropionate 51, a moderate diastereoselectivity was observed, although the yield was not satisfying. On the other hand, the reaction of 53 afforded carboxylic acid 54 having contiguous quaternary carbons in 66% yield. It should be noted that the acetoxy group could survive under the reaction conditions in stark contrast to the Ireland-Claisen rearrangement.

**Scheme 12:**
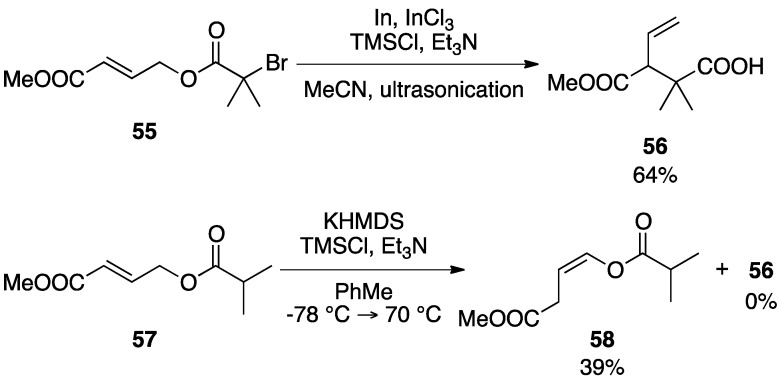
Attempted rearrangements of **55** and **57**.

The results shown in Scheme 12 reveals a marked advantage over the Ireland-Claisen rearrangement. Thus, when the reaction of **55** was performed under the above mentioned In-mediated rearrangement conditions, the rearranged product **56** was obtained in 64% yield. In contrast, the reaction of **57** with KHMDS in the presence of TMSCl and Et_3_N afforded isomer **58** in place of **56** [29,30,31].

### 3.4. Proposed Mechanism of the Indium-Mediated Reformatsky-Claisen Rearrangement

**Scheme 13:**
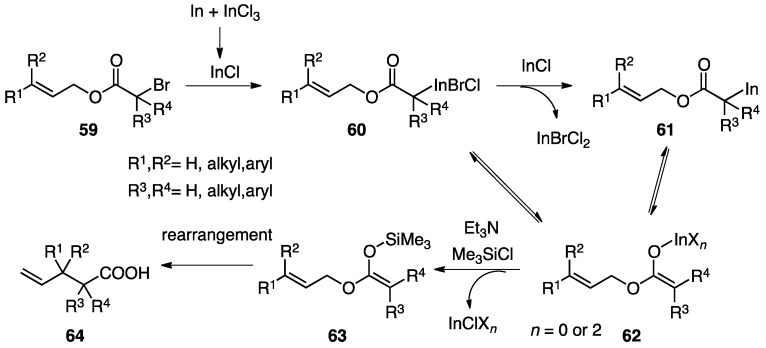
Proposed mechanism of Reformatsky-Claisen rearrangement.

As illustrated in Scheme 13, it has been reported that a mixture of In and InCl_3_ generates InCl (I) *in situ*, which readily reacts with α-bromoacetate **59** to afford α-In(III) intermediate **60** or α-In(I) **61** [27]. Both α-indium intermediates can be transformed to the In enolate **62**, which is converted to silyl ketene acetal **63** by silylation. Since no rearrangement was observed without TMSCl and Et_3_N, the direct rearrangement of In enolates seems unlikely. Finally, the rearrangement of **63** proceeds to generate the corresponding carboxylic acid **64**. The rearrangement of α-bromoisobutyrate derivatives (R_3_, R_4_ = Me) smoothly proceeded in MeCN, whereas the reaction of α-bromopropionates (R_3_ = Me, R_4_ = H) in MeCN afforded only the protonated products. These results can be rationalized as follows: compared to enolate **62** (R_3_, R_4_ = Me) derived from isobutyrate, the enolate **62** derived from the propionate derivative (R_3_ = Me, R_4_ = H) is more nucleophilic, so it easily undergoes protonation with MeCN to form a protonated product. The deuteration experiment in MeCN-d_3_ clearly supported that the rearrangement would proceed, provided that the protonation of an enolate is relatively slow.

## 4. Conclusions

The recent studies show that Zn- and In-mediated Reformatsky-Claisen rearrangements of α-haloacetate derivatives are regarded as a useful variant of the classical Ireland-Claisen rearranegements. The feasibility of these methods for base-sensitive substrates makes it complementary to the Ireland-Claisen rearrangement, and allows simple access to valuable building blocks for the synthesis of complex natural products. 
